# RISK FACTORS FOR FINANCIAL FRAUD AMONG OLDER CHINESE: AN ANALYSIS BASED ON CHARLS DATA

**DOI:** 10.1093/geroni/igae098.1397

**Published:** 2024-12-31

**Authors:** Yuanyuan Li, Yunqi Cao, Yuxia Wu, Hong Mi

**Affiliations:** Hebei University of Technology, TianJin, Tianjin, China (People’s Republic); Hebei University of Technology, TianJin, Tianjin, China (People’s Republic); Ningbo University of Technology, Ningbo, Zhejiang, China (People’s Republic); Zhejiang University, Hangzhou, Zhejiang, China (People’s Republic)

## Abstract

Financial fraud targeting older adults in China is a growing concern, resulting in both financial losses and adverse effects on their physical and mental health. Despite its prevalence, there remains a scarcity of research focused on the victims of these frauds. Leveraging the 2018 China Health and Retirement Longitudinal Study (CHARLS) database and applying structural equation modeling (SEM), this study delves into the factors that place older adults in China at risk of financial fraud, examining the issue through the lens of the victims themselves. The frameworks typically used in Western contexts like the United States and Britain only focused on individual level factors, such as age and living arrangement, physical functioning, and individual capital (i.e., levels of education, cognition, and social activities). This study integrates unique social models, including factors related to family relationships, to reflect the Chinese cultural setting. It offers a comprehensive view of the elements that heighten the risk of financial fraud among older Chinese. The analysis uncovered that factors such as age and living arrangement, physical functioning, family relationship, and individual capital play a direct role in increasing the vulnerability of older adults to financial scams. Furthermore, psychological vulnerability and material wealth were identified as mediators among physical functioning, family relationships, and fraud risks. Based upon these findings, the study proposes targeted strategies to mitigate the risk of financial fraud to include promoting healthier living habits, enhancing individual capital, and reinforcing family connections.

